# Study of the occurrence of intra-abdominal hypertension and abdominal compartment syndrome in patients of blunt abdominal trauma and its correlation with the clinical outcome in the above patients

**DOI:** 10.1186/s13017-016-0066-5

**Published:** 2016-02-11

**Authors:** Ajeet Ramamani Tiwari, Jayashri Sanjay Pandya

**Affiliations:** Department of General Surgery, Topiwala National Medical College and Bai Yamunabai Laxman Nair Charitable Hospital, Mumbai Central, 400008 India

**Keywords:** Blunt abdominal trauma, Intra-abdominal pressure, Abdominal compartment syndrome, Intra-abdominal hypertension, Bladder pressure, Non-operative management

## Abstract

**Background:**

Intra-abdominal pressure (IAP) measurements have been identified as essential for diagnosis and management of both intra-abdominal hypertension (IAH) and Abdominal compartment syndrome (ACS). It has gained prominent status in ICUs worldwide. We aimed to evaluate the utility of measurement of rise in bladder pressure to assess IAP levels in blunt abdominal trauma (BAT) patients.

**Patients and methods:**

Thirty patients of BAT with solid organ injuries were included in this study. Intra-abdominal pressure was measured through a Foleys bladder catheter throughout their stay. Bladder pressure was compared with clinical parameters like mean arterial pressures(MAP), respiratory rate(RR), serum creatinine(SC) and abdominal girth(AG) and also with outcome in terms of intervention whether operative(OI) or non-operative(NOI).

**Results:**

Bladder pressure showed significant correlation with MAP (R = −0.418; *P* = 0.022), AG (R = 0.755; *P* = 0.000), SC (R = 0.689; *P* = 0.000) and RR (R = 0.537; *P* = 0.002). Bladder pressure (R = 0.851; *P* = 0.000), SC (R = 0.625; *P* = 0.000), MAP (R = −0.350; *P* = 0.058) and maximum AG difference (R = 0.634; *P* = 0.000) showed significant correlation with intervention. In total, 17 patients (56 %) required intervention, 9 patients (30 %) underwent NOI (pigtailing or aspiration) while 8 (27 %) needed OI. More than 3 derailed parameters were associated with 100 % intervention (Mean 3.47, SD-1.23). High APACHE III score on admission (>40) was associated with increased intervention (*p* = 0.001). Intervention correlates well with Grade of injury (*p* = 0.000) and not with number of organs injured (*p* = 0.061). Blood transfusion of 2 or more units of blood was associated with increased intervention (*p* = 0.000).

**Conclusion:**

Increased bladder pressure and other clinical parameters (MAP, SC, RR and change in AG) correlates well with intervention. Elevated bladder pressure correlates well with other clinical parameters in patients with BAT. Bladder pressure, SC, MAP, RR and AG difference can be used to determine the group of patients that can be managed conservatively and those that would benefit with minimal intervention or exploration. During Non-operative management (NOM) of patients with BAT and multiple solid organ injuries, IAP monitoring may be a simple and objective guideline to suggest further intervention whether NOI or OI. Although routine bladder pressure measurements will result in unnecessary monitoring of large number of patients it is hoped that patients with IAH can be detected early and subsequent ACS with morbid abdominal exploration can be prevented. However the criterion for non-operative failure and the point of decompression needs further refinement to prevent an increase of nontherapeutic operations.

## Background

Since 19^th^ century, intra-abdominal hypertension (IAH) and abdominal compartment syndrome (ACS) have been recognized. ACS has been indicated as a complication in serious blunt abdominal trauma (BAT) for more than 50 years. It develops as a consequence of increased intra-abdominal pressure (IAP) not only in abdominal trauma, but also in intestinal obstructions with serous edema of the bowels or a chronically growing ascites [1, 2]. IAH and ACS are major factors responsible for significant morbidity and mortality among the critically ill patients and their role has been appreciated in last 15 years [3–5].

The incidence of IAH in critical care patients is reported to be 50 %, of these 50 %, 32.1 % develop IAH and 4.2 % develop ACS within their first day of ICU [6, 7]. Historically physical observation and measurement of abdominal girth were used to determine the presence of IAH. This method of measurement is inaccurate due to a high risk of variability and low reliability. A range of approaches to measure IAP include intra gastric, intra rectal, inferior vena cava and via a urinary indwelling catheter pressure monitoring systems [5, 8–11].

Continuous bladder IAP measurements are more reliable to intermittent measurements. This technique was first described by *Kron* et al. which involves placing a Foley catheter in the urinary bladder. It is considered as the ‘gold standard’ for indirect clinical measurement of IAP [12–15].

Non Operative Management (NOM) is the treatment of choice for BAT since last few decades because of increased evidence of surgery related complications [16, 17]. NOM consists of five therapeutic strategies to overcome IAH/ACS, includes evacuation of intra luminal content, evacuation of intra-abdominal space occupying lesions, improvement in abdominal wall compliance, optimization of fluid administration and tissue perfusion by serially monitoring of patients with the help of different imaging techniques [18].

Monitoring these patients during NOM requires precise clinical skills taking into account various clinical and laboratory parameters. Most of these standard clinical parameters monitored during NOM collectively help the surgeon to take decisions that are very subjective in nature. Many of these patients develop distension of abdomen following few days of trauma which might be due to trivial causes such as continued slow hemorrhage and bile leak causing peritoneal irritation and excessive fluid secretion with bowel edema. However the distension might also be due to serious complications like massive re-bleeding, delayed bowel perforation or frank biliary peritonitis. While monitoring these patients in critical care unit with parameters like pulse, blood pressure, hemogram, ABG, abdominal girth at small time intervals the surgeon always has a dilemma as to whether the deteriorating clinical condition is either due to these trivial causes or serious ones that would mandate an open exploration. Repeat imaging with CT scan might help sometimes for serious complications like delayed perforation or massive re-bleeds however they are not specific in the event of development of ACS for trivial causes. None of the above clinical parameters objectively help the surgeon to define occurrence of ACS in these patients which many times can be tackled with plain decompression via less invasive means like aspiration and pigtailing rather than ending up with morbid negative abdominal explorations.

In the quest to objectively define development of ACS in the patients of BAT managed with NOM, we serially measured their bladder pressures while they were being treated in their respective surgical units. At the end of the treatment we tried to correlate different clinical parameters and bladder pressures to propose that serial bladder pressure monitoring should be a part of clinical assessment of these patients for early diagnosis and timely intervention in the event of development of ACS.

## Methods

This was a prospective descriptive observational study carried out at our tertiary center after obtaining permission from the Institutional Ethics Committee. 30 patients of BAT with solid organ injuries were included in the study. However patients with head and spinal injury, urinary bladder injury, history of neurogenic bladder or previous bladder surgery were excluded from the study as above conditions would cause variations in bladder pressure measurements. At presentation patient’s/Patient’s legally accepted relative’s consent was obtained for inclusion into the study.

Patients were admitted in one of the 6 surgical units of the hospital depending upon the day they presented to the hospital. All patient’s APACHE III score was calculated at admission as per departmental policy and patients were managed as per surgical unit’s trauma management protocols. The common management protocol for all the 6 surgical units were initial resuscitation as per ATLS protocols, CT abdomen when vitally stable, blood, FFP and crystalloid transfusion when required, hourly monitoring of pulse, BP, CVP, respiratory rate, abdominal girth, urinary output, serum creatinine, 6 hourly monitoring of ABG, hemoglobin, hematocrit and strict immobilization for a minimum of 3 days with continued limb mobilization, compression stockings and chest physiotherapy. However the decision of operative management of these patients, who deteriorated upon a trial of NOM, was solely made by surgical unit head, after taking into account the above mentioned clinical and laboratory parameters. The bladder pressures of all the patients who fit into the inclusion criteria was monitored 6 hourly from the time of admission when the patients were catheterized. The end point was removal of Foley’s catheter either before discharge or upon death. Intra-abdominal pressure was measured through a Foleys bladder catheter using water column measurement at pubic symphysis.

At the end of the study, sequential bladder pressure was compared to the outcome in terms of intervention whether operative (OI) or non-operative (NOI) and raised bladder pressures correlated with clinical parameters like mean arterial pressures (MAP), respiratory rate (RR), serum creatinine (SC) and abdominal girth (AG). Statistical analysis was performed by software SPSS20.0. Chi square test and spearman correlation was calculated to correlate various clinical parameters with intervention and increased bladder pressure. Descriptive analysis performed to assess demographic data.

## Results

Out of the 30 patients included in the study, 28 (93 %) were males and 2 (7 %) were female patients between 18 to 60 years (mean-32, SD-12.17) of age. The minimal APACHE III score was 8 while maximum was 94 (mean-45.4, SD-22.80) at entry level. 13 (43 %) patients required no intervention and settled with conservative management while 17 (57 %) patients required intervention. Out of 17, 9 (53 %) patients underwent NOI while 8 (47 %) needed OI. The mean hospital stay was 11 days (minimum 6 and maximum 16). During hospital stay, 24 patients required blood transfusion at some point. One patient succumbed to her injuries while rest of the patients were discharged after treatment.

**Fig. 1:**
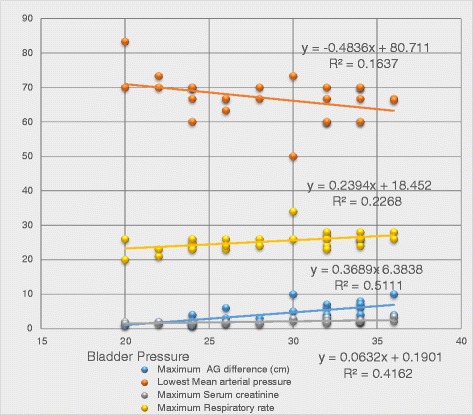
Chart showing correlation between Bladder pressure, abdominal Girth (AG) difference, mean arterial pressure, serum creatinine, respiratory rate and Intervention during the course of treatment for blunt abdominal trauma

The cut off values were, AG change of more than or equal to 3 cm from the time of admission, SC more than or equal to 2 mg/dl, MAP less than or equal to 70 mmHg, RR more than or equal to 24 per minute and bladder pressure more than or equal to 25 cm H2O as abnormal. Correlation was obtained when these parameters were compared at time of intervention. Parameters with respect to their cut off values were statistically analyzed with intervention. Bladder pressure (R = 0.851; *P* = 0.000), SC (R = 0.625; *P* = 0.000), MAP (R = −0.350; *P* = 0.058) and maximum AG difference (R = 0.634; *P* = 0.000) showed significant correlation with intervention. (Table 1, Fig. 1).

**Table 1:** Correlation between bladder pressure, abdominal Girth (AG) difference, mean arterial pressure, serum creatinine, respiratory rate and intervention during the course of treatment for blunt abdominal trauma

			Maximum bladder pressure	Intervention vs no intervention
Spearman’s rho	Maximum bladder pressure	Correlation coefficient	1.000	0.851
		Sig. (2-tailed)		.000
	Maximum AG difference (cm)	Correlation coefficient	.755	0.634
		Sig. (2-tailed)	.000	.000
	Lowest mean arterial pressure	Correlation coefficient	-.418	−0.350
		Sig. (2-tailed)	.022	.058
	Maximum serum creatinine	Correlation coefficient	.689	0.625
		Sig. (2-tailed)	.000	.000
	Maximum respiratory rate	Correlation coefficient	.537	0.445
		Sig. (2-tailed)	.002	.014
	Intervention vs no intervention	Correlation coefficient	-.851	1.000
		Sig. (2-tailed)	.000

Statistical correlation analysis of change in SC, MAP, RR and AG was performed with increase in bladder pressure. MAP (R = −0.418; *P* = 0.022), AG (R = 0.755; *P* = 0.00), SC (R = 0.689; *P* = 0.000) and RR (R = 0.537; *P* = 0.002) showed significant Spearman correlation with respect to rise in bladder pressure. More than 3 derailed parameters were associated with 100 % intervention (Mean 3.47, SD-1.23). (Table 1, Fig. 1).

Increased APACHE III score (>40) was associated with increased intervention (*p* = 0.001). Intervention correlates well with grade of injury (*p* = 0.000) and not with number of organs injured. Blood transfusion of 2 or more units of blood correlates with intervention (*p* = 0.000) (Table 2).

**Table 2:** Cross table showing relation between Apache III score, grades of injury, number of organs injured, blood transfusion and intervention

Apache III score	No intervention	Intervention	Chi square value	*P* value
<=40	11	5	11.808	0.001
>40	1	13		
Grades of injury				
1,2	8	0	16.364	0.000
3,4	4	18		
Number of organs injured				
> = 2	4	13	4.434	0.061
1	8	5		
Blood transfusion (units)				
<2	11	1	22.245	0.000
> = 2	1	17		

## Discussion

Abdominal trauma can result in the increase of IAP for a variety of reasons, including the accumulation of blood or free fluid in the peritoneal cavity, edema of the intestinal wall, retroperitoneal hematoma or abdominal packing for hemorrhage control. Therefore the continuing hepatic hemorrhage and increasing amounts of bloody ascites found in failed NOM can lead to an elevation in IAP [19]. ACS with multiple organ dysfunction is a consequence of the effects of IAH on multiple organ systems. Elevated IAP results in impaired physiology and organ functions due to the limited abdominal wall compliance [20]. In patients with severe trauma the incidence of ACS has been reported at 14 % − 15 % after damage control laparotomies. To date there are very few reports describing the changes in IAP or the development of IAH or ACS while the patients are receiving NOM after BAT [19]. NOM has been established as the treatment of choice for most of the patients with BAT with solid organ injury.

Similar to Croce et al. who reported mean transfusion requirement in first 48 h to be 1.9 units our overall mean transfusion requirement was 1.93 units however no limitation of transfusion requirement was mentioned [21]. Most authors favor the ultimate decisive factor of NOM should be hemodynamic stability of the patient. However no definitive limitation of transfusion requirement to maintain the hemodynamic stability in such patients has been documented. In our study 2 or more units of blood transfusion was associated with increased intervention. A falling hematocrit presents the surgeon with a dilemma as to whether the liver laceration is the cause of the ongoing bleeding especially in patients with multiple solid organ injuries. Although the CT scan study can objectively demonstrate the change in solid organ lesions and the amount of hemoperitoneum, this diagnostic modality is not readily available. Moreover, the hepatic and splenic lesion may be confused with the initial ingress of interstitial fluid between the lacerations [19].

Since no adverse effects of IAP below 25 cm H2O were reported this value was therefore used as a cutoff point for our study. Ray-Jade Chen et al. studied ACS in patients with hepatic injuries in BAT and using IAP of 25 cm H2O as cutoff point compared the two groups with respect to estimated liver related transfusions, PaO2/FiO2 and peritoneal signs. All patients with IAP of 25 cm of H2O or greater had exacerbation of peritoneal signs before operation and a strong correlation between the IAP value and presence of peritoneal signs was found [19]. In our study we did not use peritoneal signs as they are highly subjective and examiner dependent. It can be confused by concomitant thoraco-abdominal or pelvic injuries and variable pain responses from different points. In our study we included patients of not only hepatic injuries but other solid organ injuries due to BAT to have a more broad generalization of ACS in this group of patients as not just hepatic injuries but other solid organ injuries too are managed conservatively and have equal probability of developing ACS. This notion is supported from our finding that it is not the number of organs that are injured (*p* = 0.061) but the grade of injury of the solid organ injured (*p* = 0.000) that causes increased intervention (Table 2). Despite the popularity of NOM no known parameters can precisely reflect the ongoing hemorrhage and predict whether the hemodynamic instability is due to continued bleeding or development of ACS. Hence in our study we included simple bedside clinical parameters i.e. Bladder pressure, RR, MAP, SC and change in AG, that are routinely used to monitor these patients with BAT undergoing NOM and found that more than 3 deranged parameters was associated with 100 % intervention.

In NOM of patients with solid organ injuries the continuing hemorrhage and increasing amount of ascites (bloody or secretory) can lead to an increase in IAP. Resuscitation and edema formation after shock or relative ischemia may result in a cyclic process that perpetuates IAP elevation. Hence for aggressive resuscitation in the patients sustaining solid organ injuries the probability of hemodynamic derangement from IAP elevation is high. A further increase of the IAP will subsequently compromise the hemodynamics and result in non-operative failure. A negative abdominal exploration in these patients can be avoided if ACS due to excessive fluid can be recognized at an early stage by continuous monitoring of bladder pressures. Burch et al. developed a working grade system for ACS on the basis of urinary bladder pressure measurements. They recommended that in patients after celiotomy with bladder pressure of 26 to 35 cm H2O decompression is necessary and with bladder pressure exceeding 35 cm H2O reexploration is mandatory [22]. These findings match with our study in which Bladder pressure more than 25 cm of H2O was statistically significant when compared to time of intervention. In our study we found that more than 3 deranged clinical parameters (out of Bladder pressure, SC, RR, MAP and AG) was associated with 100 % intervention.

Ray-Jade Chen et al. described the failure of NOM in their study as those that- 1. Manifested hemodynamic derangement (systolic pressure < 90 mm Hg) despite resuscitation, or 2. Had stable hemodynamics but with an IAP of 25 cm H2O or greater. Of the 25 patients of BAT with liver injuries that Ray-Jade Chen et al. studied, 5 developed IAP greater than 25 cm H2O and underwent laparoscopy for decompression [19]. In our study we did not use laparoscopy instead decompression was achieved through pigtailing and multiple aspirations of the intra-abdominal collections in 9 patients who underwent NOI (Table 3). 19 patients(76 %) were successfully treated non operatively in Ray-Jade Chen et al. study that matched with our finding of 22 patients out of 30 (73 %).

**Table 3:** Data showing types of intervention in patients with different organ injuries

		Intervention			Total
		Operative	Non operative	None	
Solid organ injury	Liver	0	1	6	7
	Liver/Kidney	0	0	1	1
	Liver/Pancreas	0	1	0	1
	Liver/Spleen	4	5	3	12
	Liver/Spleen/Kidney	0	1	0	1
	Liver/Spleen/Pancreas	1	0	0	1
	Liver/Spleen/Pancreas/Kidney	0	1	0	1
	Spleen	3	0	3	6
Total		8	9	13	30

Of the 17 patients that required intervention 9 of them underwent NOI and the morbidity of negative abdominal exploration was avoided by simple procedures like pigtailing and aspiration (Table 3). In a study by Cheatham et.al, percutaneous catheter decompression (PCD) was compared with open abdominal decompression in treatment of IAH and ACS. PCD potentially avoided the need for subsequent open abdominal decompression in 25 of 31 patients (81 %) treated [23]. The lower mortality found in our study suggests that IAH and ACS can be managed using non-operative methods, which will prevent morbidity associated with exploratory laparotomy. However L. Pleva et al. suggested that in the event of any suspicion for acute elevation of IAP and development of ACS the performance of decompression laparotomy is indicated even with the assumption of negative preoperative finding [24]. Nevertheless identification of this group of patients by continuous bladder pressure monitoring who develop ACS due to increased fluid inside the abdominal cavity and not due to ongoing hemorrhage is essential as the morbidity of a negative exploration can be avoided by simple decompressive NOI and can buy some more time for successful completion of NOM.

Limitations of our study was non utilization of complex physiologic monitoring of hemodynamic and pulmonary dysfunction in ACS like the oxygen delivery index and peak airway pressures as Swan Ganz catheter was not routinely used in our trauma units. Also while they were receiving NOM the cardiac output index and oxygen transport variables were not measured. Our preliminary study was small and the criterion of decompression needs further evaluation.

## Conclusion

Increased bladder pressure and other clinical parameters (MAP, SC, RR and change in AG) correlates well with intervention. Elevated bladder pressure correlates well with other clinical parameters in patients with BAT. Bladder pressure, SC, MAP, RR and AG difference can be used to determine the group of patients that can be managed conservatively and those that would benefit with minimal intervention or exploration. It is the grade of injury to solid organs that determine intervention and not the number of solid organs injured. During NOM of patients with BAT and multiple solid organ injuries, IAP monitoring may be a simple and objective guideline that might be in future included in Trauma Severity Scales to suggest further intervention whether NOI or OI. Although routine bladder pressure measurements will result in unnecessary monitoring of large number of patients it is hoped that patients with IAH can be detected early and subsequent ACS with morbid abdominal exploration can be prevented. However the criterion for non-operative failure and the point of decompression needs further refinement to prevent an increase of nontherapeutic operations.
